# Probing Water State during Lipidic Mesophases Phase Transitions

**DOI:** 10.1002/anie.202110975

**Published:** 2021-10-22

**Authors:** Yang Yao, Sara Catalini, Bence Kutus, Johannes Hunger, Paolo Foggi, Raffaele Mezzenga

**Affiliations:** ^1^ Department of Health Sciences and Technology ETH Zürich Schmelzbergstrasse 9 8092 Zürich Switzerland; ^2^ Department of Materials ETH Zürich Wolfgang-Pauli-Strasse 10 8093 Zürich Switzerland; ^3^ European Laboratory for Non-Linear Spectroscopy, LENS Via Nello Carrara 1 50019 Florence Italy; ^4^ Department of Chemistry University of Perugia Via Elce di Sotto 8 06123 Perugia Italy; ^5^ Max Planck Institute for Polymer Research Ackermannweg 10 55128 Mainz Germany

**Keywords:** dielectric spectroscopy, interfaces, lipidic mesophase, phase transitions, water dynamics

## Abstract

We investigate the static and dynamic states of water network during the phase transitions from double gyroid ($Ia\bar{3}d$ ) to double diamond ($Pn\bar{3}m$ ) bicontinuous cubic phases and from the latter to the reverse hexagonal (*H*_II_) phase in monolinolein based lipidic mesophases by combining FTIR and broadband dielectric spectroscopy (BDS). In both cubic(s) and *H*_II_ phase, two dynamically different fractions of water are detected and attributed to bound and interstitial free water. The dynamics of the two water fractions are all slower than bulk water due to the hydrogen‐bonds between water molecules and the lipid's polar headgroups and to nanoconfinement. Both FTIR and BDS results suggest that a larger fraction of water is hydrogen‐bonded to the headgroup of lipids in the *H*_II_ phase at higher temperature than in the cubic phase at lower temperature via H‐bonds, which is different from the common expectation that the number of H‐bonds should decrease with increase of temperature. These findings are rationalized by considering the topological ratio of interface/volume of the two mesophases.

## Graphical abstract

We study the phase transitions from cubic to reverse hexagonal phase in lipidic mesophase at molecular level. The dynamics of both water and lipids, as well as the hydrogen bond network of confined water were monitored during the phase transition. The results demonstrate clear dynamical difference between bound and interstitial water in both phases.

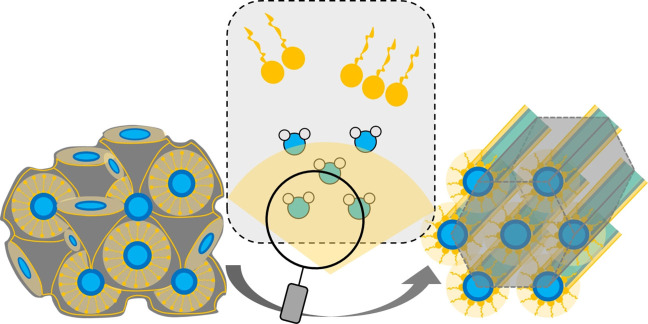

Liquid water is the prerequisite for life, and it is the unique medium for biological processes.^[1]^ In biological systems, the static and dynamic properties of water are essential to the structure, stability and function of biomolecules such as nucleic acids, proteins, and lipids.^[2]^ Without water, biomolecules lose their biological functions and may hardly be considered as true biomolecules.^[3]^ At the same time, the crowded and confining environment provided by biomolecules and other small molecules also influence the hydrogen‐bond network of water molecules.[^4^, ^5^, ^6^] Water under confinement behaves differently from the bulk in many aspects. In hard confinement systems such as nanoporous alumina,^[7]^ hollow silica,^[8]^ and mesoporous silica,[^9^, ^10^, ^11^] the nucleation mechanism of water changes and acquires a confinement‐dependent behavior. In extreme cases where the pore diameter *d*<26 Å, water can be supercooled below the homogeneous nucleation temperature.^[12]^ Two dynamically different fractions of water were detected and assigned to the interfacial water close to pore walls and the inner water, respectively.^[12]^ Beyond hard confinement, the water state in soft confinement systems has tremendous importance in biological processes such as enzymatic reactions.^[13]^ One reported soft confining media is given by reverse micelles stabilized by dioctyl sodium sulfosuccinate (AOT) where a water pool is surrounded by the polar headgroups of the surfactant.[^14^, ^15^] The diameter of water pools is adjusted by varying the amount of water.[^14^, ^16^] Both experiments and molecular dynamics simulations showed two types of water inside the reverse micelles: a slow interfacial water and a bulk‐like core.[^16^, ^17^, ^18^]

Lipidic lyotropic liquid crystals (LLCs), also known as lipidic mesophases, forming by the self‐assembly of lipids in water, offer an alternative confining media, and an ideal biocompatible platform for protein crystallization,[^19^, ^20^, ^21^] drug delivery,^[22]^ and enzymatic reactions.^[23]^ Contrary to the AOT and other water‐in‐oil systems, LLC provides continuous/bicontinuous water channels which enables the diffusion and transportation of substrates and products. A variety of liquid crystal structures can be obtained depending on the amphiphilic properties of the lipid, the degree of hydration, and the temperature. These structures include reverse micelle (*L*_2_), lamellar phase (*L*_α_), bicontinuous cubic phases (gyroid, $Ia\bar{3}d$ diamond, $Pn\bar{3}m$ , and primitive, $Im\bar{3}m$ ), and reverse hexagonal phase (*H*_II_). In bicontinuous cubic phases, the midplane of the lipid bilayer is arranged on a triply periodic minimal surface, where the mean curvature at each point is zero.[^24^, ^25^, ^26^, ^27^] As for the *H*_II_ phase, relatively straight circular cylinders are arranged in a 2D array: each cylinder is surrounded by a layer of lipid molecules with hydrophobic tails pointing outward the water struts.^[28]^ The phase structure and curvature of LLC play an essential role in its application, especially in the field of drug delivery.[^29^, ^30^] Besides, the physical behavior of water confined in different mesophases may also vary from each other, though the state of water under soft confinement of the complex LLCs is yet an emerging field on itself. Recently, the dynamics and crystallization of water confined in the simplest *L*_α_ phase in a phytantriol‐based LLC was reported in the subzero temperature region.^[31]^ Broadband dielectric spectroscopy (BDS) was used to obtain the dynamics of confined water from the relaxation of dipole moments under electric fields.^[32]^ The relaxation times of both bound water close to the lipid headgroup and inner interstitial free water were detected within *L*_α_ phase, where the reorientational dynamics of bound water was found two to three orders of magnitude slower than that of interstitial water.^[31]^

Herein, we combine different techniques to investigate the state of both water and lipids in monolinolein‐based lipidic mesophase system during the phase transitions from double gyroid ($Ia\bar{3}d$ ) to double diamond ($Pn\bar{3}m$ ) bicontinuous cubic phases and from the latter to the reverse hexagonal (*H*_II_) phase at molecular level, where the dynamic processes of the lipid molecules during the phase transition are obtained combining small angle X‐ray scattering (SAXS) and BDS. The association of polar headgroups show longer relaxation time in the *H*_II_ phase compared to the cubic phase, despite the former is forming at much higher temperature. Furthermore, since hydrogen bonds (H‐bonds) are the most important type of interactions among water molecules and between water and biomolecules, we provide deep insights into the water state under confinement LLCs by a combination of Fourier‐transform infrared spectroscopy (FTIR) and BDS. Among the results we uncover, lies the evidence that the H‐bond network of confined water is more disrupted in the *H*_II_ phase than in the cubic phase. Furthermore, a slightly larger amount of bound water was detected in the *H*_II_ phase compared to $Ia\bar{3}d$ phase, despite the higher temperature of the *H*_II_. This result is apparently against both common understanding based on the vanishing of H‐bonds with increase of temperature and self‐consistent field theories implementing reversible H‐bonds.^[33]^ We show, however, that these results can be understood by considering the specific interfacial area, i.e., the total interfacial area within a mesophase repeat unit volume, which we extract by small angle X‐rays scattering and structural models for the various mesophases, yielding a higher specific area for the *H*_II_ phase compared to $Ia\bar{3}d$ phase, and thus a higher propensity to promote bound water in the *H*_II_ phase.

The mesophase structures of monolinolein‐water systems in the temperature range from 25–67 °C were obtained from SAXS (Figure 1). Two samples were chosen according to the phase diagram,^[34]^ with 30 wt % and 35 wt % of water, respectively. Both samples experience the phase transition from $Pn\bar{3}m$ to *H*_II_ phase. The SAXS spectra of the sample with 30 wt % water at room temperature show Bragg reflections with relative positions in ratios of $\sqrt{6}$ :$\sqrt{8}$ :$\sqrt{14}$ :$\sqrt{16}$ :$\sqrt{20}$ :$\sqrt{22}$ , which reflects a cubic $Ia\bar{3}d$ phase (Figure 1 a). The $Ia\bar{3}d$ phase remained stable up to 31 °C and then transformed into cubic $Pn\bar{3}m$ phase with the relative positions in ratios of $\sqrt{2}$ :$\sqrt{3}$ :$\sqrt{4}$ :$\sqrt{6}$ :$\sqrt{8}$ :$\sqrt{9}$ . The pure $Pn\bar{3}m$ phase remained up to 49 °C then gradually transformed into a reversed hexagonal phase (*H*_II_) at 67 °C with the relative positions in ratios of $\sqrt{1}$ :$\sqrt{3}$ :$\sqrt{4}$ . The cubic $Pn\bar{3}m$ phase coexisted with the *H*_II_ phase in the phase transition temperature range of 49–67 °C. The dimension of water confined in LLCs is calculated from the structure parameters of different phases as a function of temperature. The calculation is described in the supporting information. Figure 1 b and c shows the temperature dependence of water channel diameter (*D*_water_) and lipid length (*L*_lip_), respectively. Within the same phase, *D*_water_ and *L*_lip_ decrease with the increase of temperature. During the phase transition from cubic $Ia\bar{3}d$ phase to cubic $Pn\bar{3}m$ phase, the continuous decrease of both *D*_water_ and *L*_lip_ is observed with the increase of temperature. However, during the phase transition from $Pn\bar{3}m$ to *H*_II_, *D*_water_ shows a discontinuous increase while *L*_lip_ exhibits a discontinuous decrease, which indicates that the hydrophobic tail is (in average) more densely packed in *H*_II_ phase. To further confirm the observation, the SAXS spectra of Di65‐W35 with higher water content (35 wt %) is applied (Figure 1 d). The phase transition from $Pn\bar{3}m$ to *H*_II_ again occurs in the temperature range of 49–67 °C. As in the case of the sample with 30 wt % water, *D*_water_ shows a discontinuous increase while *L*_lip_ has a discontinuous decrease from $Pn\bar{3}m$ to *H*_II_ (Figure 1 e, f).

**Figure 1:**
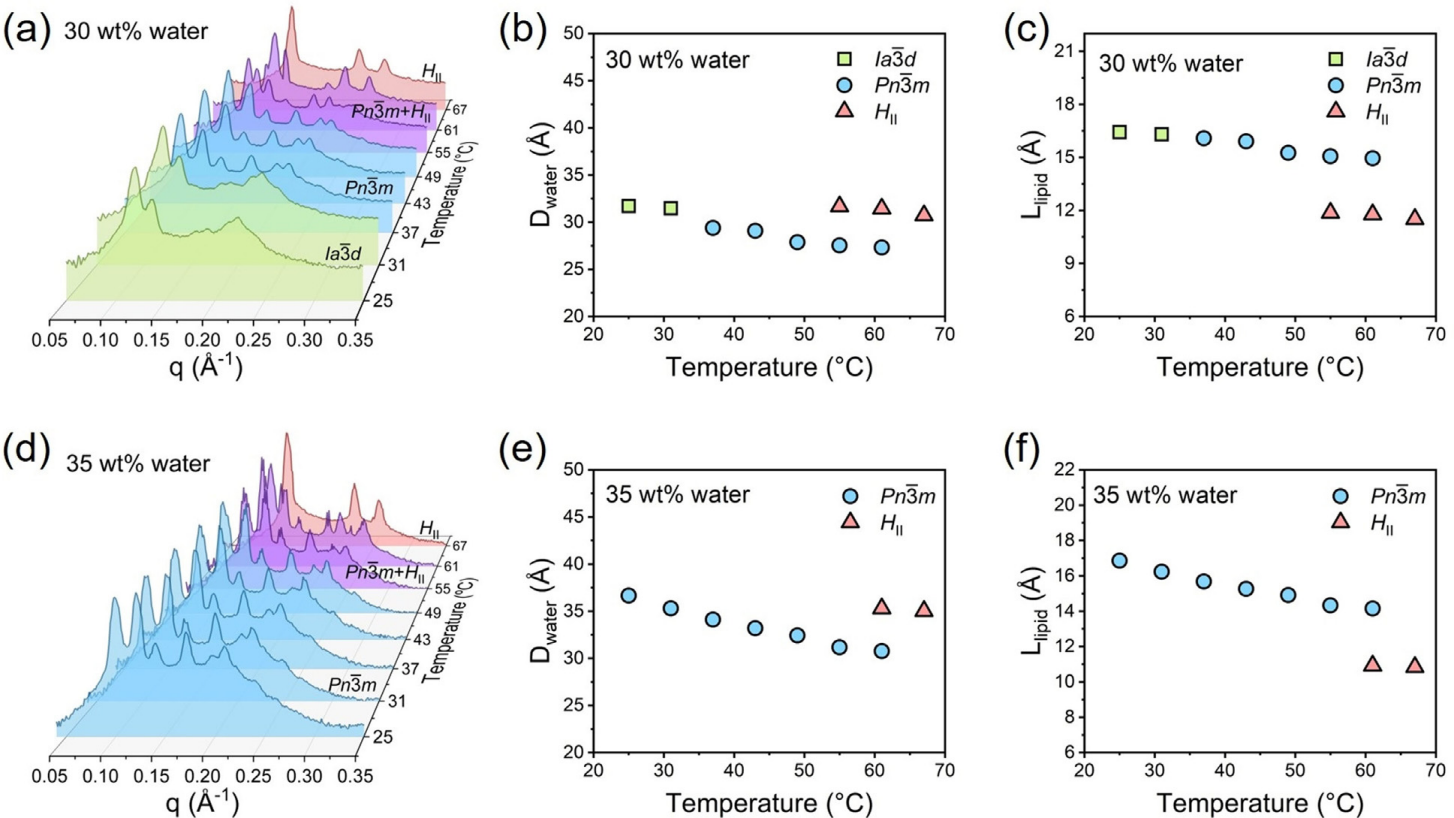
a) SAXS profiles of LLC with 30 wt % water. Water channel diameter (*D*_water_) b) and of lipid chain length (*L*_lip_) c) of the $Ia\bar{3}d$ , $Pn\bar{3}m$ , and *H*_II_ mesophases in LLC with 30 wt % water. d) SAXS profiles of LLC with 35 wt % water. Water channel diameter (*D*_water_) e) and of lipid chain length (*L*_lip_) f) of the $Pn\bar{3}m$ , and *H*_II_ mesophases in LLC with 35 wt % water.

To explore the phase transition at molecular level, we studied the dynamics of lipid during the phase transition by broadband dielectric spectroscopy (BDS). The 3D plots of BDS spectra in the form of tan*δ* (tan*δ*=*ϵ*′′/*ϵ*′) versus frequency (10^−2^–10^6^ Hz) of monolinolein‐water samples with different water content measured in the temperature range of 25–70 °C are shown in Figure 2 a–c. In the spectra of pure monolinolein, two processes can be observed (Figure 2 a). The relaxation times for each process as a function of temperature are obtained from the fitting of the BDS spectra using two Havriliak–Negami (HN) models. The result is shown in Figure 2 d. Both the two processes are slower than the usual α‐process associated with the segmental dynamics of the lipid molecules (Figure S1). The relaxation time of the faster process 2 is close to the supramolecular dynamics that originates from the association of headgroups (e.g. dimers or trimers) with a Debye distribution of relaxation times.^[35]^ The slower process 1 is assigned to the chain dynamics of lipid molecule, due to the low dielectric strengths (Figure S2). Both process 1 and 2 are related to the global dynamics of the lipid molecule in the pure monolinolein sample, thus, they both show a kink at the melting temperature (*T*_m_) (Figure S3).

**Figure 2:**
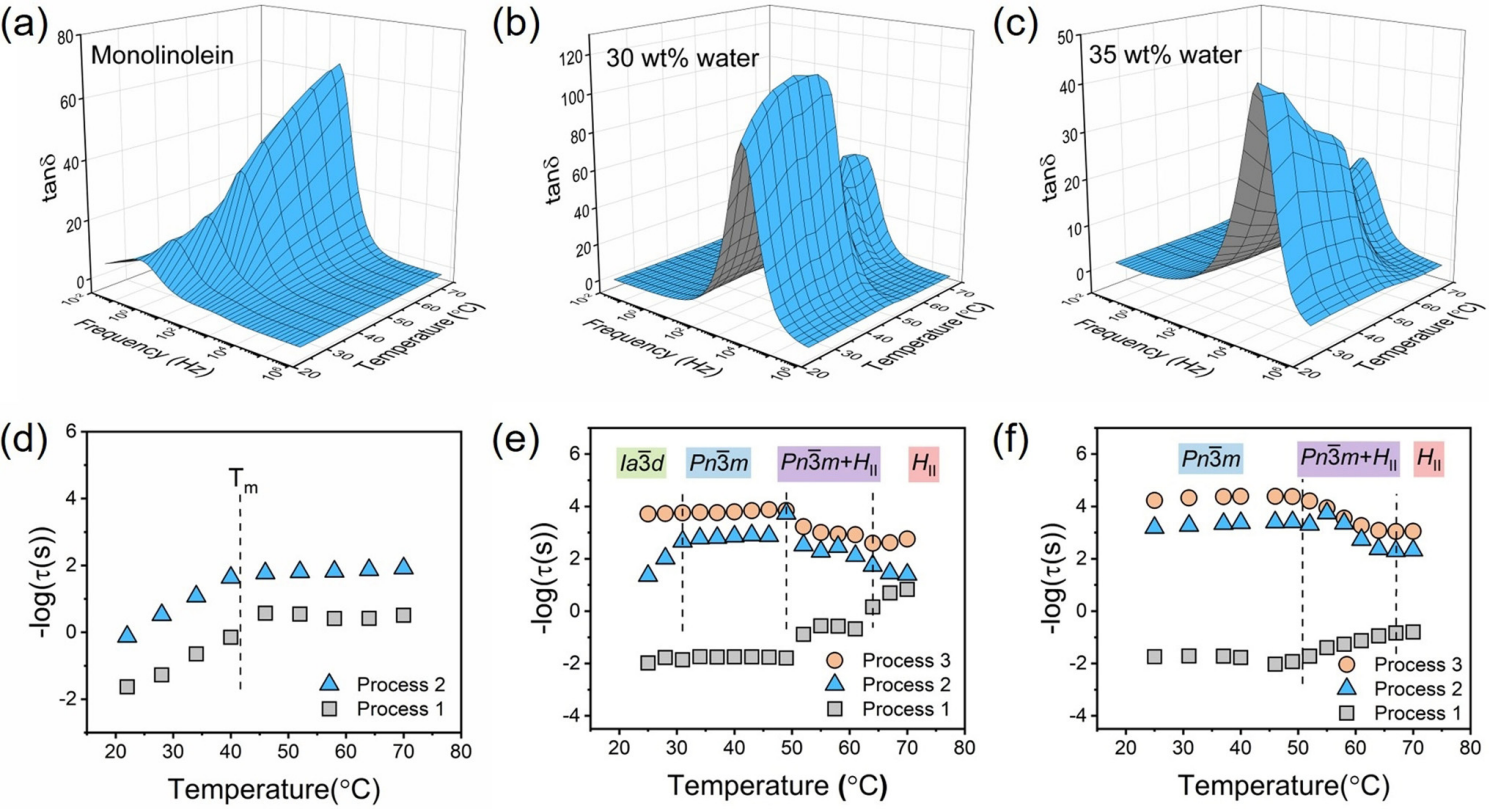
3D representation of tan*δ* as a function of frequency in a range of temperatures for a) pure monolinolein, b) LLC with 30 wt % water, and c) LLC with 35 wt % water. Summary of relaxation times obtained from HN fittings of BDS tan*δ* spectra for d) pure monolinolein, e) LLC with 30 wt % water, and f) LLC with 35 wt % water. Dash lines indicate the melting temperature of monolinolein (obtained from DSC, Figure S3), the phase transition temperatures obtained from SAXS for different samples, respectively.

The BDS spectra become more diverse with the increase of water content. A significant fluctuation is observed in the 3D plot of the sample with 30 wt % and 35 wt % water in the temperature range of the phase transition from $Pn\bar{3}m$ to *H*_II_ (49–67 °C) (Figure 2 b, c). The plots of relaxation times show three relaxation processes with different temperature dependencies. At 25 °C and above, the dynamics of water occurs in GHz frequency range which will be discussed later. Regarding the frequency range below 1 MHz, the three processes observed are all from the dynamics of lipid molecules. The slowest process 1 appears in the frequency window of 10^−2^–100 Hz (Figure 2 e) and shows a shorter relaxation time in *H*_II_ than in $Pn\bar{3}m$ phase. This has certain similarity to the finding reported in a former rheology study of the same system^[34]^ where both the storage (G′) and loss moduli (G′′) moduli are higher in $Pn\bar{3}m$ than in *H*_II_ phase, reflecting that the system is with higher mobility in *H*_II_ phase than in $Pn\bar{3}m$ . Besides process 1, two faster processes (process 2 and 3) are observed in the plots of relaxation times of the two samples. Both the two processes show an increase in the relaxation time upon the phase transition from $Pn\bar{3}m$ to *H*_II_ phase (Figure 2 e, f). The fitting parameters are summarized in the supporting information (Figure S2). Process 3 is close to the Debye relaxation where *α*=1, *γ*=1, indicating that the process originates from the headgroup association and reorientation.[^32^, ^35^] On the other hand, process 2 follows a Cole‐Cole (CC) relaxation where α<1, *γ*=1. The CC relaxation is reported to arise from the interaction of the relaxing dipole moment with the underlying physical matrix.^[36]^ In this case, process 2 is assigned to the interaction of polar headgroups within the matrix of LLCs. Thus, it shows a slower dynamics but with similar temperature dependence as process 3 since the relaxing of headgroups are confined by the LLC matrix. In general, the dynamics of both process 2 and process 3 from headgroups are faster in the $Pn\bar{3}m$ than in the *H*_II_ phase, which is related to the topological difference between the two phases.

The static state of H‐bond network of water confined in monolinolein‐water mesophase is investigated by FTIR in the temperature range of 25–70 °C. Within the spectral window from 2600 to 3100 cm^−1^, both the information from the structure of the water network by the OH‐stretching band, and the conformation of the lipid's hydrophobic tails by the CH_2_ symmetric and asymmetric stretching signals can be obtained (Figure 3 a). In general, the H‐bond strength is correlated with the vibrational frequency: more strongly H‐bonded water molecules exhibit lower OH stretching frequencies. To capture changes in water H‐bonded structure, we model the OH stretching band with three Gaussian functions, which are sometimes ascribed to ice‐like, distorted, and free configuration of water network (Figure 3 b). The ice‐like configuration at lower frequency (ca. 3200 cm^−1^) arises from the ordered H‐bond contribution where water molecules are tetrahedrally coordinated. The distorted configuration refers to the “closed” water structure (ca. 3400 cm^−1^) where H‐bonds are partially distorted. The free configuration arises from the OH groups weakly stabilized by H‐bond interactions (ca. 3600 cm^−1^). It is noteworthy that all these configurations of the OH oscillators are in dynamic equilibrium with each other and are generated transiently during the H‐bond reorganization of the water network.[^37^, ^38^] However, the fraction of different configurations varies as a function of temperature and in different aqueous environment. In pure water and aqueous solutions, with the increase of temperature, the increased thermal motions progressively disrupt the tetrahedral order of water network, and the ratio of ice‐like component (*A*_ice‐like_/*A*_tot_) decreases linearly. Meanwhile, the ratios of the distorted (*A*_distorted_/*A*_tot_) and free (*A*_free_/*A*_tot_) components increase linearly with increasingtemperature, which reflects a less structured water network upon heating.^[39]^

**Figure 3:**
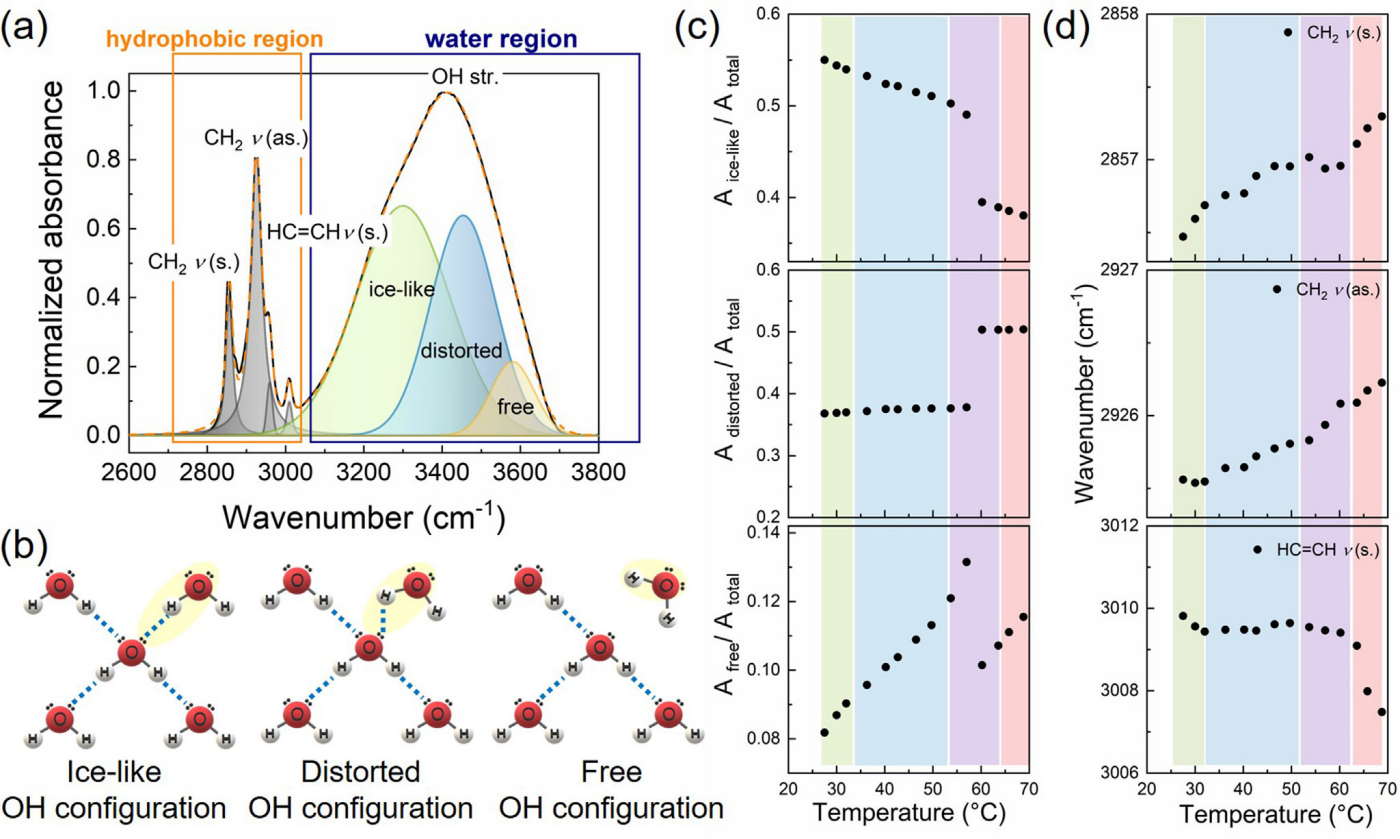
a) Fitting example of FTIR spectra in the region of 2600–3800 cm^−1^ of LLC with 30 wt % water at 28 °C. b) schematic representation of three different types of OH configurations in water network. c) fraction of ice‐like, distorted, and free OH stretching components as a function of temperature obtained from fitting the FTIR spectra of LLC with 30 wt % water. d) wavenumber trend of the CH_2_ symmetric, CH_2_ asymmetric, and HC=CH stretching bands as a function of temperature of LLC with 30 wt % water. The colored areas correspond to the phase structures obtained from SAXS: $Ia\bar{3}d$ (green), $Pn\bar{3}m$ (blue), *H*_II_ (red), mixture of $Pn\bar{3}m$ and *H*_II_ (purple).

In LLC, the polar headgroups of lipids interact with water molecules and influences the OH‐stretching bands of water network. The effect of phase transition from $Pn\bar{3}m$ to *H*_II_ phase on the network of confined water was obtained from the temperature dependence of the fractions of the different OH configurations (Figure 3 c). Apart from the linear change of the three OH stretching components within the same phase, a notable discontinuity is clearly observed at 58 °C, the phase transition temperature. It is clearly seen that beyond this temperature there is a decrease in ice‐like and free OH fractions with a simultaneous increase in the distorted OH fraction, suggesting an increased disorder in the water network in the *H*_II_ phase as compared to the $Pn\bar{3}m$ phase. The hypothetical scenario that explains the different fractions of water components between the two phases is that, along with increased disorder in the water network, a larger amount of water molecules confined in *H*_II_ phase is bonded to the headgroups of lipids than in $Pn\bar{3}m$ phase despite the slight increase of the water channel diameter in *H*_II_ phase. The newly formed H‐bonds between water molecules and the polar headgroup in *H*_II_ phase disturb the tetrahedral order of the water network. As a result, the ice‐like and the free component largely decreases. On the other hand, the distorted component increases which is likely due to the increased amount of H‐bonds between confined water and polar headgroups. This finding is further confirmed by the analysis of polar headgroup region of the lipids (Figure S3) and the BDS of water (Figure 4 d) and will be addressed later below by topological structural models.

**Figure 4:**
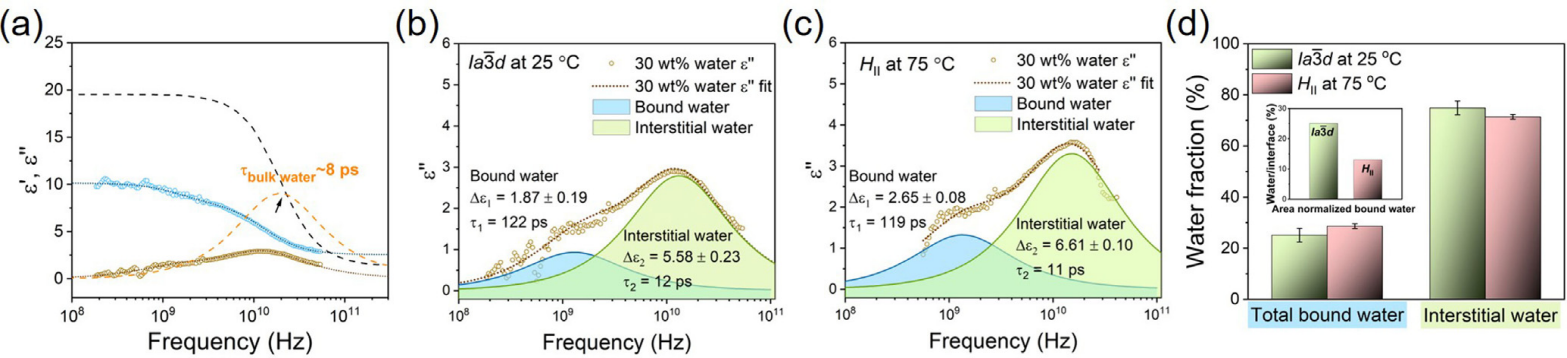
a) Dielectric permittivity (*ϵ*′) and dielectric loss (*ϵ*′′) spectra at GHz frequencies of LLC with 30 wt % water at 25 °C. Open points refer to experimental data (*ϵ*′ in blue and in *ϵ*′′ orange), dotted line is the fitting of the data according to Debye model, assuming two relaxation modes. For comparison, the spectrum of bulk water at 25 °C (dashed lines, taken from the literature and multiplied by 0.25^[44]^) is also shown. Panels (b) and (c) depict the decomposition of the *ϵ*′′ spectra into the two Debye relaxations for the LLC with 30 wt % water at b) 25 °C and c) 75 °C, as obtained from the fit. d) The comparison of the fractions of bound and interstitial water in the LLC with 30 wt % water between the $Ia\bar{3}d$ phase at 25 °C and the *H*_II_ phase at 75 °C. The insert of (d) is the bound water fraction normalized by the interface/volume between the two phases.

Figure 3 d shows the temperature dependence of CH_2_ symmetric and asymmetric stretching resulting from the hydrophobic tails of the lipid. The wavenumbers of both the two signals undergo a blue shift with increase of temperature, indicating an enhanced chain mobility with high vibrational energy. On the contrary, the HC=CH stretching undergoes a red shift with the increase of temperature at the phase transition from $Pn\bar{3}m$ to *H*_II_ phase. This could be caused by a lengthening of the HC=CH bond that decreases the oscillator vibrational frequency or by dense packing of the lipid's tails in *H*_II_ phase,[^37^, ^40^] as was seen in SAXS result.

We further explored the dynamic state of water confined in LLC during the phase transition by BDS at GHz frequencies. First, the spectrum of 30 wt % water sample is compared to that of bulk water at 25 °C (Figure 4 a). The spectrum of bulk water (multiplied by 0.25) is dominated by one Debye‐type relaxation, giving rise to a dispersion in the relative permittivity (*ϵ*′) as well as a peak in the dielectric loss (*ϵ*′′) spectrum, centred at ≈20 GHz. Conversely, the spectrum of 30 wt % water (in the $Ia\bar{3}d$ phase at 25 °C) can be well described using two Debye relaxations and the overall relaxation strength (amplitude) is weaker than for bulk water. This reduced dielectric strength can be largely explained by the reduced volume concentration of water. Yet, the detected relaxation strength at GHz frequencies is even lower than a volume/mass scaled spectrum of pure water (Figure 4 a), which may be explained by reduced dipolar correlations.[^41^, ^42^]

The faster Debye relaxation was obtained at 13 GHz (mode 2) at 25 °C. The relaxation time (τ_2_) increases from ≈8 in pure water to 12 ps in $Ia\bar{3}d$ phase, suggesting that the reorientational dynamics are ≈1.5‐fold slower in $Ia\bar{3}d$ phase than in bulk water. Based on the similar relaxation times, we assign the corresponding relaxation process in $Ia\bar{3}d$ phase to the reorientational relaxation of bulk‐like water (interstitial) water. Additionally, we find a low‐frequency Debye‐mode (mode 1, centred at ≈1.3 GHz) for 30 wt % water sample (Figure 4 b). In the *H*_II_ phases we find—similar to the $Ia\bar{3}d$ phase, two dynamically different water fractions (Figure 4 c), which is different from the assignment to the three fractions of water confined in mesophase in a former study.^[43]^ According to the spatial distribution of water molecules, the slower process is assigned to the relaxation of bound water molecules residing in close proximity of the polar headgroup of lipid, whereas the faster, bulk‐like process is assigned to the reorientation of interstitial water located further away from the headgroup. The dynamics of bound water is slower by nearly one order of magnitude than interstitial water in both phases (*τ*_1_≈119–122 ps vs. *τ*_2_≈11–12 ps).

In general, dielectric relaxation is a thermally activated process, thus, it is surprising that the *τ*_1_ and *τ*_2_ relaxation times remain essentially unaltered upon phase transition, despite the 50 °C increase in temperature. This strongly suggests that the acceleration of relaxation dynamics is counteracted by the enhancement of water confinement due to phase transition.

The proportion of bound water and interstitial water in each phase can be obtained from the dielectric relaxation strength (Δ*ϵ*) of the processes. Based on the Debye‐Onsager model, the dielectric strength is proportional to the number density of the dipoles (*N*), the square of the corresponding dipole moments (*μ* ^2^) as well as the dipole‐dipole correlation factor (*g*), accounting for the correlated motions of relaxing species (Δ*ϵ*≈*g* *Nμ* ^2^). Assuming that the dipole‐dipole correlations of both states of liquid water are identical, the Δ*ϵ* values of bound and interstitial water are comparable and their relative fraction can be obtained as Δ*ϵ*_1/2_/(Δ*ϵ*_1_+Δ*ϵ*_2_). As shown in Figure 4 d, the proportion of bound water is slightly higher in the *H*_II_ phase than in the $Ia\bar{3}d$ phase, which is consistent with the FTIR findings.

This result hinting at a larger amount of bound water in the *H*_II_ phase than in the $Ia\bar{3}d$ phase is at first surprising since the number of H‐bonds is well known to decrease with increase in temperatures. To rationalize these results, we calculate the specific area, that is the ratio of lipid‐water‐interface/volume for both *H*_II_ and $Ia\bar{3}d$ phase, for the sample with 30 wt % water (see supporting information for topological models). For *H*_II_ phase, the interface/volume can be calculated according to the geometric structure. For bicontinuous $Ia\bar{3}d$ cubic phase, the interface/volume ratio can be calculated according to triply periodic minimal surface (TPMS), where the water and lipid are considered as two distinct domains.[^25^, ^45^, ^46^] The calculation is explained in supporting information.

The ratio of interface/volume values for *H*_II_ and $Ia\bar{3}d$ phase is estimated to be 0.40 and 0.18 nm^−1^, respectively. The interface/volume in the *H*_II_ phase at 67 °C is more than double that of the $Ia\bar{3}d$ phase at 25 °C. This explains the higher driving force towards bound water in the *H*_II_ phase; However, the bound water is only 16 % higher in the *H*_II_ phase at 75 °C than in $Ia\bar{3}d$ phase 25 °C, due to the different temperatures considered. The structural and temperature effects can be disentangled if the bound water fraction is further normalized by the interface/volume value of the two phases, which yields a normalized bound water fraction much smaller in the *H*_II_ than in the $Ia\bar{3}d$ phase (the insert of Figure 4 d). This indicates a large decrease in the number of H‐bonded water molecules per head groups in the *H*_II_ phase at high temperature.

The state of water depends strongly on the geometry of the structure of host lipidic lyotropic liquid crystal. We explored the phase transition from cubic to reverse hexagonal (*H*_II_) phase by looking into the static and dynamic states of water and lipids by combining several techniques. The phase transition temperature was confirmed by SAXS. In the dynamic study by BDS, three processes from lipids were detected in monolinolein‐water mesophase: headgroup association, interaction of polar headgroups with the matrix of LLCs, and a global dynamics. The lipid headgroups in *H*_II_ phase presented slower dynamics than in cubic phase despite the higher temperature due to the topological difference between the two phases. With respect to confined water, two dynamically different fractions of water (both slower than the bulk water) were detected both in cubic phase and in *H*_II_ phase. Additionally, the dielectric intensity provided the ratio between bound and interstitial water. Compared to cubic phase, a slightly larger amount of water molecules were bonded to the headgroups of lipid in the *H*_II_ phase. This finding was also confirmed by the FTIR results, where a stronger structure‐breaking on water H‐bond network was observed in *H*_II_ phase compared to that in $Pn\bar{3}m$ phase. These apparently counter‐intuitive results are rationalized by the topological ratio between interface/volume in the two phases. This analysis shows that the *H*_II_ phase, with a larger specific interfacial area per unit volume promotes a larger driving force for binding of water molecules to lipid heads; however, the normalized number of water molecules that H‐bonded to the headgroup is simultaneously decreased in the *H*_II_ phase compared to the $Ia\bar{3}d$ phase due to the high temperature, an effect that was disentangled from the structural driving force by a careful topological analysis. The results from this study may open a new strategy in the study of order‐order transitions in lipidic mesophases and other complex structured fluids, by simply following the evolution on the state of water within these systems.

## Conflict of interest

The authors declare no conflict of interest.

## Supporting information

As a service to our authors and readers, this journal provides supporting information supplied by the authors. Such materials are peer reviewed and may be re‐organized for online delivery, but are not copy‐edited or typeset. Technical support issues arising from supporting information (other than missing files) should be addressed to the authors.

Supporting Information

## References

[1] L. J. Rothschild , R. L. Mancinelli , Nature 2001, 409, 1092–1101. 11234023 10.1038/35059215

[2] D. Laage , T. Elsaesser , J. T. Hynes , Chem. Rev. 2017, 117, 10694–10725. 28248491 10.1021/acs.chemrev.6b00765 PMC5571470

[3] Y. Levy , J. N. Onuchic , Annu. Rev. Biophys. Biomol. Struct. 2006, 35, 389–415. 16689642 10.1146/annurev.biophys.35.040405.102134

[4] J. C. Rasaiah , S. Garde , G. Hummer , Annu. Rev. Phys. Chem. 2008, 59, 713–740. 18092942 10.1146/annurev.physchem.59.032607.093815

[5] F. Martelli , J. Crain , G. Franzese , ACS Nano 2020, 14, 8616–8623. 32578978 10.1021/acsnano.0c02984

[6] M. Tros , L. Zheng , J. Hunger , M. Bonn , D. Bonn , G. J. Smits , S. Woutersen , Nat. Commun. 2017, 8, 904. 29026086 10.1038/s41467-017-00858-0 PMC5714959

[7] Y. Suzuki , H. Duran , M. Steinhart , M. Kappl , H.-J. Butt , G. Floudas , Nano Lett. 2015, 15, 1987–1992. 25686014 10.1021/nl504855z

[8] Y. Yao , P. Ruckdeschel , R. Graf , H.-J. Butt , M. Retsch , G. Floudas , J. Phys. Chem. B 2017, 121, 306–313. 27960260 10.1021/acs.jpcb.6b11053

[9] B. Grünberg , T. Emmler , E. Gedat , I. Shenderovich , G. H. Findenegg , H. H. Limbach , G. Buntkowsky , Chem. Eur. J. 2004, 10, 5689–5696. 15470692 10.1002/chem.200400351

[10] L. Liu , S.-H. Chen , A. Faraone , C.-W. Yen , C.-Y. Mou , Phys. Rev. Lett. 2005, 95, 117802. 16197049 10.1103/PhysRevLett.95.117802

[11] M. Sattig , S. Reutter , F. Fujara , M. Werner , G. Buntkowsky , M. Vogel , Phys. Chem. Chem. Phys. 2014, 16, 19229–19240. 25096474 10.1039/c4cp02057j

[12] Y. Yao , V. Fella , W. Huang , K. A. Zhang , K. Landfester , H.-J. Butt , M. Vogel , G. Floudas , Langmuir 2019, 35, 5890–5901. 30946592 10.1021/acs.langmuir.9b00496

[13] N. E. Levinger , Science 2002, 298, 1722–1723. 12459570 10.1126/science.1079322

[14] T. K. De , A. Maitra , Adv. Colloid Interface Sci. 1995, 59, 95–193.

[15] M. Kotlarchyk , J. S. Huang , S. H. Chen , J. Phys. Chem. 1985, 89, 4382–4386.

[16] B. Baruah , J. M. Roden , M. Sedgwick , N. M. Correa , D. C. Crans , N. E. Levinger , J. Am. Chem. Soc. 2006, 128, 12758–12765. 17002370 10.1021/ja0624319

[17] D. E. Moilanen , E. E. Fenn , D. Wong , M. D. Fayer , J. Chem. Phys. 2009, 131, 014704. 19586114 10.1063/1.3159779 PMC2721765

[18] A. M. Dokter , S. Woutersen , H. J. Bakker , Proc. Natl. Acad. Sci. USA 2006, 103, 15355–15358. 17028175 10.1073/pnas.0603239103 PMC1592463

[19] A. Zabara , J. T. Y. Chong , I. Martiel , L. Stark , B. A. Cromer , C. Speziale , C. J. Drummond , R. Mezzenga , Nat. Commun. 2018, 9, 544. 29416037 10.1038/s41467-018-02996-5 PMC5803273

[20] M. Caffrey , V. Cherezov , Nat. Protoc. 2009, 4, 706–731. 19390528 10.1038/nprot.2009.31 PMC2732203

[21] E. Landau , J. P. Rosenbusch , Proc. Natl. Acad. Sci. USA 1996, 93, 14532–14535. 8962086 10.1073/pnas.93.25.14532 PMC26167

[22] S. Assenza , R. Mezzenga , J. Chem. Phys. 2018, 148, 054902. 29421907 10.1063/1.5019224

[23] T. Zhou , J. J. Vallooran , S. Assenza , A. Szekrenyi , P. Clapés , R. Mezzenga , ACS Catal. 2018, 8, 5810–5815.

[24] D. M. Anderson , S. M. Gruner , S. Leibler , Proc. Natl. Acad. Sci. USA 1988, 85, 5364–5368. 3399497 10.1073/pnas.85.15.5364 PMC281757

[25] N. Garti , P. Somasundaran , R. Mezzenga , Self-assembled supramolecular architectures: lyotropic liquid crystals, Wiley, Hoboken, 2012, pp. 3–17.

[26] W. Mickel , G. E. Schröder-Turk , K. Mecke , Interface Focus 2012, 2, 623–633. 24098847 10.1098/rsfs.2012.0007 PMC3438574

[27] S. Hyde , S. Andersson , K. Larsson , Z. Blum , T. Landh , S. Lidin , B. W. Ninham , The Language of Shape: The Role of Curvature in Condensed Matter: Physics, Elsevier, Amsterdam, 1997.

[28] I. Amar-Yuli , E. Wachtel , E. B. Shoshan , D. Danino , A. Aserin , N. Garti , Langmuir 2007, 23, 3637–3645. 17328564 10.1021/la062851b

[29] P. B. Ishai , D. Libster , A. Aserin , N. Garti , Y. Feldman , J. Phys. Chem. B 2009, 113, 12639–12647. 19722513 10.1021/jp901987p

[30] R. Ghanbari , S. Assenza , R. Mezzenga , J. Chem. Phys. 2019, 150, 094901. 30849886 10.1063/1.5080929

[31] Y. Yao , T. Zhou , R. Färber , U. Grossner , G. Floudas , R. Mezzenga , Nat. Nanotechnol. 2021, 16, 802—810. 33941918 10.1038/s41565-021-00893-5

[32] F. Kremer , A. Schönhals , Broadband dielectric spectroscopy, Springer Science & Business Media, Cham, 2002, pp. 17–23.

[33] W. B. Lee , R. Mezzenga , G. H. Fredrickson , Phys. Rev. Lett. 2007, 99, 187801. 17995439 10.1103/PhysRevLett.99.187801

[34] R. Mezzenga , C. Meyer , C. Servais , A. I. Romoscanu , L. Sagalowicz , R. C. Hayward , Langmuir 2005, 21, 3322–3333. 15807570 10.1021/la046964b

[35] C. Gainaru , R. Meier , S. Schildmann , C. Lederle , W. Hiller , E. Rössler , R. Böhmer , Phys. Rev. Lett. 2010, 105, 258303. 21231631 10.1103/PhysRevLett.105.258303

[36] Y. E. Ryabov , Y. Feldman , Physica A 2002, 314, 370–378.

[37] A. Nilsson , A. Holmgren , G. Lindblom , Chem. Phys. Lipids 1994, 71, 119–131. 10.1016/0009-3084(94)90003-5 8194158

[38] J. Eaves , J. Loparo , C. J. Fecko , S. Roberts , A. Tokmakoff , P. Geissler , Proc. Natl. Acad. Sci. USA 2005, 102, 13019–13022. 16135564 10.1073/pnas.0505125102 PMC1201598

[39] P. Sassi , M. Paolantoni , R. S. Cataliotti , F. Palombo , A. Morresi , J. Phys. Chem. B 2004, 108, 19557–19565.

[40] A. Nilsson , A. Holmgren , G. Lindblom , Chem. Phys. Lipids 1994, 69, 219–227. 8194158 10.1016/0009-3084(94)90003-5

[41] A. Rahman , F. H. Stillinger , J. Chem. Phys. 1972, 57, 4009–4017.

[42] J. C. R. Reis , T. Iglesias , Phys. Chem. Chem. Phys. 2011, 13, 10670–10680. 21544282 10.1039/c1cp20142e

[43] W. Wachter , G. Trimmel , R. Buchner , O. Glatter , Soft Matter 2011, 7, 1409–1417.

[44] S. Schrödle, PhD thesis, University of Regensburg (Germany), **2005**.

[45] D. C. Turner , Z.-G. Wang , S. M. Gruner , D. A. Mannock , R. N. McElhaney , J. phys. II 1992, 2, 2039–2063.

[46] C. Speziale , L. S. Manni , C. Manatschal , E. M. Landau , R. Mezzenga , Proc. Natl. Acad. Sci. USA 2016, 113, 7491–7496. 27313210 10.1073/pnas.1603965113 PMC4941478

