# Supplementary material for: Probing Water State during Lipidic Mesophases Phase Transitions
Source: Angew Chem Int Ed Engl. 2021 Oct 22;60(48):25274–80. doi: 10.1002/anie.202110975 (PMC9298331; doi:10.1002/anie.202110975)
Supplement: Supplementary file 1 — Supporting Information [file ANIE-60-25274-s001.pdf]

Angewandte Chemie
Eine Zeitschrift der Gesellschaft Deutscher Chemiker

Supporting Information

Probing Water State during Lipidic Mesophases Phase Transitions

*Yang Yao, Sara Catalini, Bence Kutus, Johannes Hunger, Paolo Foggi, and Raffaele Mezzenga**

anie_202110975_sm_miscellaneous_information.pdf

Supporting Information
Table of Contents

I. Experimental Procedures 2
II. Calculation of water channel diameter (D_{water}) and lipid length (L_{lip}) in LLCs. 3
III. Calculation of interface/volume ratio in Ia3d and H_{II} phase. 4
IV. Broadband dielectric spectroscopy (BDS). 5
V. Differential scanning calorimetry (DSC). 6
VI. Fourier-transform infrared spectroscopy (FTIR). 7
References 8

I. Experimental Procedures

Materials. Preparation of lipidic lyotropic liquid crystal. Dimodan U/J was provided by Danisco, Denmark, as a generous gift and was used as received. Dimodan is a commercial grade of monolinolein that contains > 98 wt% monoglyceride. The molecular structure of monolinolein is shown in Figure S4a. The Milli-Q water was obtained from a Millipore purification system operating at 18.2 MΩ·cm.

Preparation of monolinolein-water mesophase. The monolinolein and water were weighed and mixed in a glass vial. The glass vial was tightly sealed with Teflon tape. The two components were stirred in the glass vials at 90 ºC for 12 min to be mixed uniformly. Subsequently, the sample was transformed and stored at room temperature for 1 day before use. The weight fraction of water was precisely controlled by measuring the weight before and after the preparation.

Small angle X-ray scattering (SAXS). SAXS measurements for monolinolein-water mesophases were performed using a Brucker AXS Micro X-ray. The operation voltage and the filament current of the microfocused X-ray source is 50 kV and 100 μA, respectively. The measurements were performed with Cu Kα radiation (λ = 1.5418 Å) collimated by a 2D Kratky collimator and the spectra were collected by a Pilatus 100K detector. The modulus of the scattering vector was calibrated using silver behenate. The samples were sealed into glass capillaries with diameter of 0.15 mm to prevent the evaporation of water. Measurements were performed every 6 ºC from 25 to 67 ºC. The samples were equilibrated for 40 min before the measurement at each temperature.

Broadband dielectric spectroscopy (BDS). The broadband dielectric measurement in the frequency range from 10^{-2} to 10^6 Hz were performed on a Novocontrol Alpha frequency analyser covering the frequency range from 0.01 to 3×10^6 Hz. A parallel plate geometry was used with a top electrode of 20 mm and bottom one of 40 mm in diameter. The mesophase sample was sandwiched between the two electrodes and the sample thickness (~50 μm) was adjusted by Teflon spacers. The isothermal measurements were made on heating from 25 to 70 ºC every 3 ºC. The complex dielectric permittivity ε^*, $\varepsilon^* = \varepsilon' - i\varepsilon''$ (ε' is the real part, and ε'' is the imaginary part), is a function of frequency f, temperature T, and pressure P, $\varepsilon^* = \varepsilon^*(f, T, P)$. Several relaxation processes contribute to ε^* in the BDS spectra of monolinolein-water mesophase; thus, we used a summation of contributions empirically described by the equation of Havriliak and Negami (HN):

$$\varepsilon^*_{HN}(\omega, T) = \varepsilon_\infty(T) + \sum_{k=1}^{l} \frac{\Delta\varepsilon_k(T)}{\left[1+\left(i\omega\tau_{HN,k}(T)\right)^{\alpha_k}\right]^{\gamma_k}} + \frac{\sigma_0(T)}{i\varepsilon_f\omega} \quad (1)$$

where k indicates the process under investigation, $\Delta\varepsilon_k(T)$ is the relaxation strength, $\tau_{HN,k}$ is the characteristic relaxation time, α_k, γ_k ($0 < \alpha$, $\alpha\gamma \leq 1$) describe the symmetrical and asymmetrical broadening of the distribution of relaxation times and ε_∞ is the dielectric permittivity at the limit of high frequencies. At lower frequencies, ε'' rises due to the conductivity ($\varepsilon'' = \sigma/(\omega\varepsilon_f)$, where σ is the dc conductivity and ε_f the permittivity of free space). The conductivity contribution has also been taken into account during the fitting process. The relaxation times at maximum loss (τ_{max}) were obtained from τ_{HN} following the transformation[1]:

$$\tau_{max,k} = \tau_{HN,k} \sin^{-1/\alpha}\left(\frac{\pi\alpha}{2(1+\gamma)}\right) \sin^{1/\alpha}\left(\frac{\pi\alpha\gamma}{2(1+\gamma)}\right) \quad (2)$$

The loss tangent functions (tanδ) were fitted using eqs 1, and 2. However, the frequencies of the maximum peak position are subtly shifted compared to the direct representation of the dielectric losses (ε''). In the case of single Debye process with no dc conductivity

$$tan\delta = \frac{\varepsilon''}{\varepsilon'} = \frac{\Delta\varepsilon\tau\omega}{\Delta\varepsilon+\varepsilon_h(1+(\omega\tau)^2)} = \frac{\tau\omega}{1+\frac{\varepsilon_h}{\Delta\varepsilon}(1+(\omega\tau)^2)} \quad (3)$$

where ε_h is the high frequency limit of the dielectric permittivity. Eq3 leads to a peak maximum when $\omega\tau = (1 + \Delta\varepsilon/\varepsilon_h)^{1/2}$.[2]

Complex permittivity spectra at GHz frequencies were recorded using an Anritsu Vector Network Analyzer (model MS4647A). The spectra in frequency range from 0.05 to 50 GHz was obtained using a frequency domain reflectometer, equipped with a coaxial open-ended probe based on 1.85 mm connector.[3] To calibrate the setup before each measurement, air, conductive silver paint, and Milli-Q water were used as standards. To avoid water evaporation, the sample vial was sealed with rubber. The measurements were done at 25 and 75 ºC.

Fourier-transform infrared spectroscopy (FTIR). The transmission FTIR measurements were performed on a Varian 640 FTIR spectroscopy. The samples were sealed between two CaF_2 windows, and the thickness was kept by a 6 μm spacer. Spectra from 1000 cm^{-1} to 4000 cm^{-1} were acquired by collecting and averaging 64 scans with a resolution of 4 cm^{-1}. The background was measured and subtracted automatically. Measurements were performed every 3 ºC from 28 to 70 ºC. The samples were equilibrated for 20 min before the measurement at each temperature.

II. Calculation of water channel diameter (D_{water}) and lipid length (L_{lip}) in LLCs.

The lattice parameter (a) for LLCs was calculated as following:
For $Ia\bar{3}d$ phase, $a = \frac{2\pi}{q^*}\sqrt{6}$
For $Pn\bar{3}m$ phase, $a = \frac{2\pi}{q^*}\sqrt{2}$
where q^* is the first peak in the SAXS profile.
For H_{II} phase, the in-plane parameter (a) and longitudinal lattice parameter (c) can be obtained from the distance (d_{hkl}) between the reflecting planes with Miller indexes of (hkl):

$$\frac{1}{d_{hkl}} = \sqrt{\frac{4}{3}\frac{(h^2+hk+k^2)}{a^2} + \frac{l^2}{c^2}} \quad (4)$$

For columnar hexagonal phase where the lattices are isotropic along the longitudinal phase, $l = 0$. Taking $d_{hkl} = 2\pi/q^*$, eq (4) can be written as:

$$a = \frac{4\pi}{q^*\sqrt{3}}\sqrt{(h^2 + hk + k^2)} \quad (5)$$

Then a can be calculated based on the q^* value of the first reflection peak with (hkl) = (100).
For bicontinuous cubic phases, the lipid length (L_{lipid}) can be calculated by the following equation

$$\varphi_{lipid} = 2A_0\left(\frac{L_{lipid}}{a}\right) + \frac{4}{3}\pi\chi\left(\frac{L_{lipid}}{a}\right)^3 \quad (6)$$

where φ_{lipid} is the volume fraction of lipid, A_0 is the minimal surface in a unit cell, χ is the Euler-Poincare characteristic.
For $Ia\bar{3}d$ phase, $A_0 = 3.091$, $\chi = -8$;
For $Pn\bar{3}m$ phase, $A_0 = 1.919$, $\chi = -2$.
The water channel radius (r) is estimated as following:
For $Ia\bar{3}d$ phase, $r = 0.248a - L_{lipid}$.
For $Pn\bar{3}m$ phase, $r = 0.391a - L_{lipid}$.
Then the water channel diameter $D_{water} = 2r$.
For H_{II} phase, the water channel radius is calculated as following:

$$r = a(1-\varphi_{lipid})^{0.5}\left(\frac{\sqrt{3}}{2\pi}\right)^{0.5} \quad (7)$$

Then, along a center-to-center edge of the repeat unit, we have

$$r = \frac{a-2L_{lipid}}{2} \quad (8)$$

where L_{lipid} is the length of the unfrustrated lipid chain.

III. Calculation of interface/volume ratio in *Ia*$\bar{3}$*d* and H_{II} phase.

For H_{II} phase, the interface/volume can be calculated according to the geometric structure:

$$interface/volume = \frac{3*2\pi r*H}{A_{H_{II}}*H} \quad (9)$$

where r is water channel radius, H is the parametric height of reverse columnar hexagonal lattice, $A_{H_{II}}$ is the cross-sectional area of the hexagonal lattice ($A_{H_{II}} = 6*\frac{\sqrt{3}}{4}*\left(2r+2L_{lipid}\right)^2$, L_{lipid} is the lipid length). The r and L_{lipid} values in H_{II} phase obtained from SAXS at 67 °C are 1.5 and 1.2 nm, respectively.
For bicontinuous $Ia\bar{3}d$ cubic phase, the interface/volume ratio can be calculated according to triply periodic minimal surface (TPMS), where the water and lipid are considered as two distinct domains. First, the lipid length (L_{lipid}) is calculated following Turner et al.[4]:

$$\varphi_{lipid} = 2A_0\left(\frac{L_{lipid}}{a}\right) + \frac{4}{3}\pi\chi\left(\frac{L_{lipid}}{a}\right)^3 \quad (10)$$

where φ_{lipid} is the volume fraction of lipid, a is the lattice parameter ($Ia\bar{3}$d, $a = \frac{2\pi}{q^*}\sqrt{6}$), A_0 is the minimal surface in a unit cell, and χ is the Euler-Poincare characteristic ($Ia\bar{3}$d, A_0 = 3.091, χ = -8).
Then the ratio of interface/volume can be calculated by:

$$interface/volume = \frac{A(\xi)}{a^3} \quad (11)$$

where $A(\xi) = A_0a^2 + 2\pi\xi^2$ is the interface per repeat unit according to Anderson et al[5, 6], $\xi = L_{lipid}$. The a and L_{lipid} values in $Ia\bar{3}d$ phase obtained from SAXS at 25 °C are 12.9 and 1.6 nm, respectively.

IV. Broadband dielectric spectroscopy (BDS).

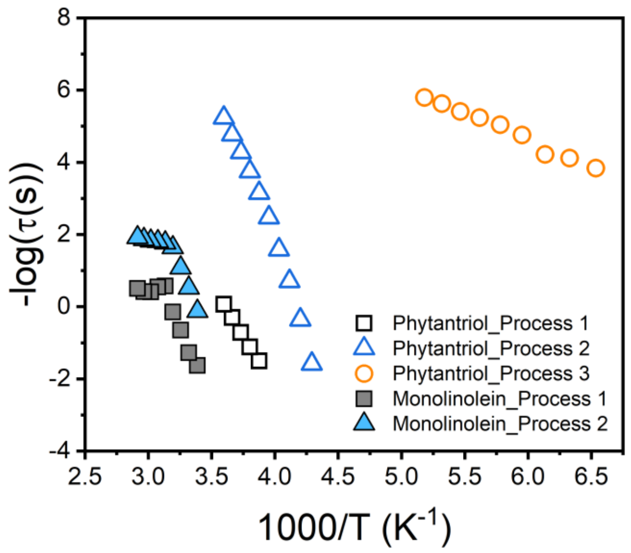

Figure S1. Comparison of relaxation times between pure monolinolein and pure phytantriol obtained from HN fitting.

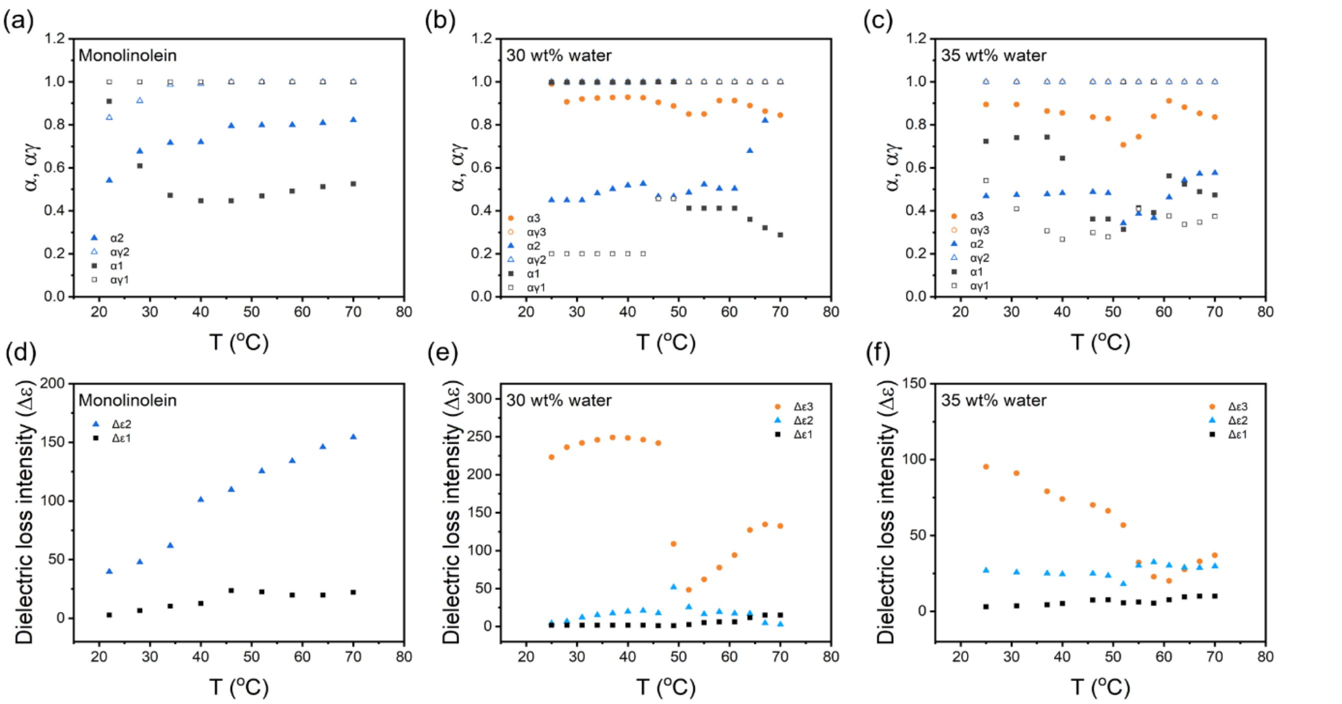

Figure S2. α, αγ parameters obtained from fitting of dielectric spectra for (a) monolinolein, (b) 30 wt% water, and (c) 35 wt% water and dielectric loss intensity obtained from fitting of dielectric spectra for (d) monolinolein, (e) 30 wt% water, and (f) 35 wt% water.

V. Differential scanning calorimetry (DSC).

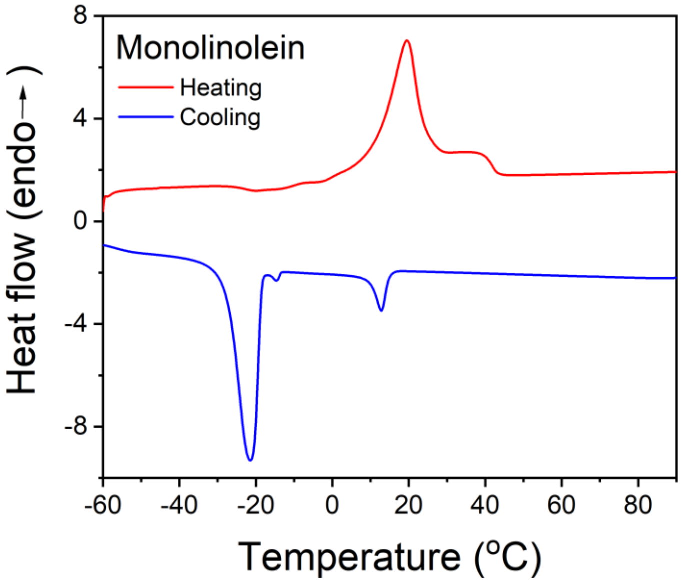

Figure S3. DSC heating and cooling curves of monolinolein, obtained with the rate of 10 °C/min.

VI. Fourier-transform infrared spectroscopy (FTIR).

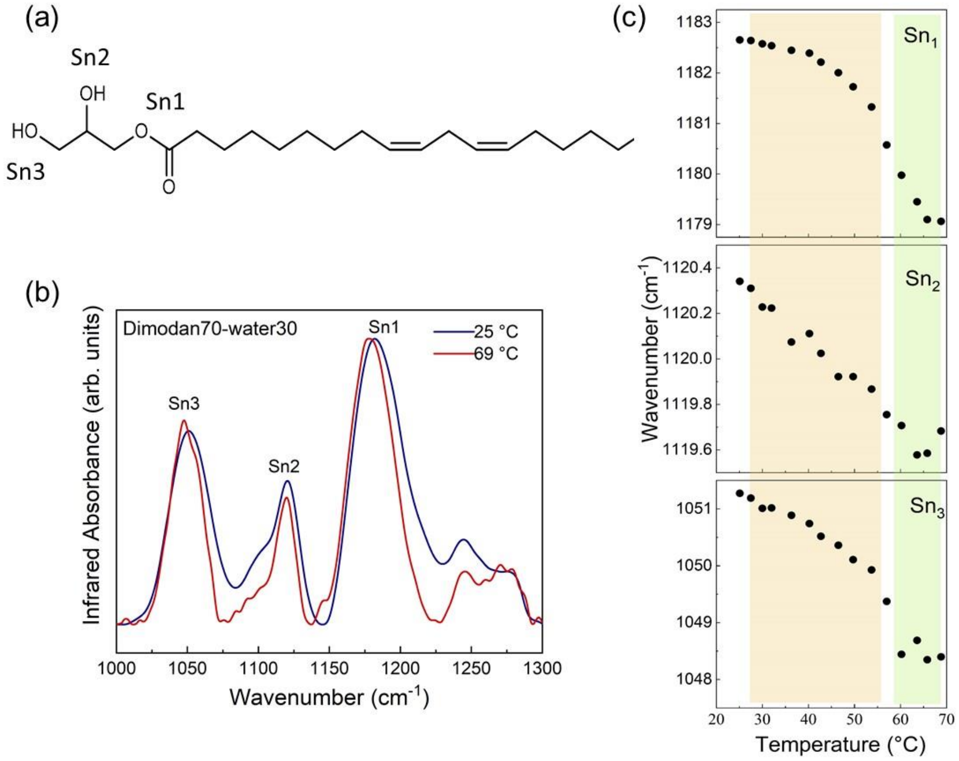

Figure S4. (a) Molecular structure of monolinolein with the label of Sn1, Sn2, and Sn3. (b) Comparison of FTIR spectra in the head group region (1000-1300 cm^{-1}) between 25 ºC and 69 ºC, and (c) wavenumber trend of the Sn1, Sn2 and Sn3 bands as a function of temperature.

The signals at 1050 cm^{-1} (Sn_3) and 1120 cm^{-1} (Sn_2) are generated by the $\mathrm{C-OH}$ stretching vibrational motion associated to the monoglyceride's headgroup that are directly involved in the estabilishment of hydrogen bonds with the sourronding water molecules. The peak at 1180 cm^{-1} (Sn_1) is assigned to the $\mathrm{CO-O-C}$ antisymmetric stretching (Figure S4). All the signals associated to the polar head undergo a red shift as a function of the temperature increases. It is known from SAXS experiment that in the cubic phases, the water channels diameter decreases with increase of temperature. Thus, the red shift of the head group stretching suggests that the water network inside the channels became more distorted and the water molecules are pushed at the water-lipid interface in the direction of the polar heads. This would involve a change in the shape of the OH stretching band in favor of the contribution arising from the "closed" water structure (ca. 3600 cm^{-1}) where H-bonds are partially distorted (Figure 3d of the manuscript). This vision is compatible with the not marked shift to lower frequencies of the signals associated with the polar heads. Furthemore, a marked change of the trend occurs at the phase trasition from $Pn\bar{3}m$ to H_{II} phase at about 58 °C. This output is also in agreement with the marked drop observed in the $A_{ice-like}/A_{tot}$ data trend at the phase transition temperature instead of the classical linear T-dependence of neat water (phenomenon commented in the manuscript).

References

[1] F. Kremer, A. Schönhals, *Broadband dielectric spectroscopy*, Springer Science & Business Media, **2002**.
[2] V. Raicu, Y. Feldman, *Dielectric relaxation in biological systems: Physical principles, methods, and applications*, Oxford University Press, USA, **2015**.
[3] W. Ensing, J. Hunger, N. Ottosson, H. Bakker, *J. Phys. Chem. C* **2013**, *117*, 12930-12935.
[4] D. C. Turner, Z.-G. Wang, S. M. Gruner, D. A. Mannock, R. N. McElhaney, *J. phys., II* **1992**, *2*, 2039-2063.
[5] D. M. Anderson, S. M. Gruner, S. Leibler, *Proc. Natl. Acad. Sci. U.S.A.* **1988**, *85*, 5364-5368.
[6] C. Speziale, L. S. Manni, C. Manatschal, E. M. Landau, R. Mezzenga, *Proc. Natl. Acad. Sci. U.S.A.* **2016**, *113*, 7491-7496.
